# 

**Published:** 1814-01

**Authors:** 


### THE MEDICAL AND PHYSICAL JOURNAL.

102416
War
35-V
40

CONDUCTED BY

SAMUEL FOTHERGILL, M.D.

OF THE ROYAL COLLEGE OF PHYSICIANS; PHYSICIAN TO THE ASYLUM FOR FEMALE ORPHANS; AND TO THE WESTMINSTER GENERAL, AND THE WESTERN, DISPENSARIES.

AND

JOHN WANT, Esq.

VOL. XXXI.

FROM DECEMBER TO JUNE, 1814.

Et quoniam variant morbi, variabimus artes;

Mille mali species, mille salutis erunt.

LIBRARY
5137

*LONDON:*

PRINTED FOR THE PROPRIETORS, BY J. ADLARD, 23, BARTHOLOMEW-CLOSE, AND 39, DUKE-STREET, SMITHFIELD; AND SOLD BY J. SOUTER, NO. 1, PATERNOSTER-ROW.

1814.

### MONTHLY CATALOGUE OF MEDICAL BOOKS.

THE Accoucheur's Vade Mecum; being the substance of a Course of Lectures on Midwifery delivered at the Westminster Lying-in Institution, Queen-Square. By Joseph Hopkins, Surgeon to his Royal Highness the Duke of Kent, &c. &c. 12mo. boards, 6s.

An Inquiry into the present State of the Medical Profession in England; containing an Abstract of all the Acts and Charters granted to Physicians, Surgeons, and Apothecaries: and a comparative View of the Profession in Scotland, Ireland, and on the Continent of Europe, &c. &c. By Robert Masters Kerrison, Member of the Royal College of Surgeons, &c. &c. 8vo. boards, 5s.

Researches into the Physical History of Man. By James Cowles Prichard, M.D. F.L.S. &c. &c. 8vo. boards, 16s.

### *TO CORRESPONDENTS.*

*Want of room has obliged us to defer the insertion of a considerable portion of Dr. Yeats's interesting Case, together with several other Communications, till next month; amongst them are Papers from Mr. C. E. Lucas; Salus Publicus; Juvenis; Pacificus; B; &c.*

